# Dynamically-Driven Enhancement of the Catalytic Machinery of the SARS 3C-Like Protease by the S284-T285-I286/A Mutations on the Extra Domain

**DOI:** 10.1371/journal.pone.0101941

**Published:** 2014-07-18

**Authors:** Liangzhong Lim, Jiahai Shi, Yuguang Mu, Jianxing Song

**Affiliations:** 1 Department of Biological Sciences, Faculty of Science, National University of Singapore, Singapore, Republic of Singapore; 2 School of Biological Sciences, Nanyang Technological University, Singapore, Republic of Singapore; King's College, London, United Kingdom

## Abstract

Previously we revealed that the extra domain of SARS 3CLpro mediated the catalysis via different mechanisms. While the R298A mutation completely abolished the dimerization, thus resulting in the inactive catalytic machinery, N214A inactivated the enzyme by altering its dynamics without significantly perturbing its structure. Here we studied another mutant with S284-T285-I286 replaced by Ala (STI/A) with a 3.6-fold activity increase and slightly enhanced dimerization. We determined its crystal structure, which still adopts the dimeric structure almost identical to that of the wild-type (WT), except for slightly tighter packing between two extra-domains. We then conducted 100-ns molecular dynamics (MD) simulations for both STI/A and WT, the longest reported so far for 3CLpro. In the simulations, two STI/A extra domains become further tightly packed, leading to a significant volume reduction of the nano-channel formed by residues from both catalytic and extra domains. The enhanced packing appears to slightly increase the dynamic stability of the N-finger and the first helix residues, which subsequently triggers the redistribution of dynamics over residues directly contacting them. This ultimately enhances the dynamical stability of the residues constituting the catalytic dyad and substrate-binding pockets. Further correlation analysis reveals that a global network of the correlated motions exists in the protease, whose components include all residues identified so far to be critical for the dimerization and catalysis. Most strikingly, the N214A mutation globally decouples this network while the STI/A mutation alters the correlation pattern. Together with previous results, the present study establishes that besides the classic structural allostery, the dynamic allostery also operates in the SARS 3CLpro, which is surprisingly able to relay the perturbations on the extra domain onto the catalytic machinery to manifest opposite catalytic effects. Our results thus imply a promising avenue to design specific inhibitors for 3CL proteases by disrupting their dynamic correlation network.

## Introduction

In 2002, severe acute respiratory syndrome (SARS) suddenly broke out in China and then rapidly spread to 32 countries, resulting in ∼8500 infections and over 900 deaths (http://www.who.int/csr/sars/en/). It is the first emerging infectious disease of the 21st century and was caused by a coronavirus termed SARS-CoV. Although now SARS appears to be contained, new coronaviruses have been detected, which may cause great threats to the human health. For example, since the appearance of a new coronavirus termed Middle East respiratory syndrome coronavirus (MERS-CoV) in April 2012, it has caused 207 confirmed cases, out of which 84 died (http://www.who.int/csr/disease/coronavirus_infections/en/index.html). More importantly, so far neither a vaccine nor an efficacious therapy has been available for them. Therefore, it remains highly demanded to develop strategies to design potential therapeutic agents against SARS- and other CoVs.

Among the known RNA viruses, coronaviruses are enveloped, positive-stranded ones with the largest single-stranded RNA genome (27–31 kilobases). The large replicase gene encodes two viral polyproteins, namely pp1a (486 kDa) and pp1ab (790 kDa), which have to be processed into active subunits for genome replication and transcription by two viral proteases [1], [2], namely the papain-like cysteine protease (PL2pro) and 3C-Like protease (3CLpro), also known as main protease (Mpro). Previously, SARS 3CLpro has been extensively characterized to be a key target for development of antiviral therapies. The coronavirus 3CLpro is so named to reflect the similarity of its catalytic machinery to that of the picornavirus 3C proteases [1]–[3]. Noticeably, both 3C and 3CL-Like proteases utilize the two-domain chymotrypsin fold to host the complete catalytic machinery, which is located in the cleft between domains I and II. Intriguingly, however, in the coronavirus 3CLpro, a ∼100-residue helical domain was evolutionarily acquired at its C-terminus [1]–[4]. Moreover, unlike 3C protease, only the homodimeric form is catalytically competent for the CoV 3CLpro [1], [4], [5]–[24]. After intense studies, now it has been clear that both chymotrypsin fold and extra domain are critical for dimerization.

We were particularly interested in understanding the role of the extra domain and thus initiated a domain dissection study on SARS 3CLpro immediately after the SARS outbreak in Singapore [6]. The results revealed that although the catalytic fold and extra domain could fold independently, the catalytic fold alone was monomeric and almost inactive. This indicates that the extra domain plays a key role in maintaining the dimerization, thus mediating the catalysis [6]. Therefore, we further conducted a systematic mutagenesis study which led to identification of the extra-domain residues critical for both dimerization and catalysis [16]. Interestingly, we found that the residues important for catalysis and dimerization constitute a nano-channel, which are composed of residues from both catalytic and extra domains [16]. Moreover, we determined the high-resolution structure of R298A, a monomeric mutant triggered by a point mutation on the extra domain, in which the most radical changes have been found within the catalytic machinery [22]. R298A adopts a completely collapsed and inactivated catalytic machinery which is structurally distinguishable from that in wild-type (WT) enzyme; with a short 3_10_-helix formed by residues Ser139-Phe140-Leu141 within the oxyanion-binding loop [22]. Remarkably, the collapsed catalytic machinery observed in R298A appears to represent a universal inactivated state intrinsic to all inactive enzymes as the same collapsed machinery was found in other monomers triggered by the mutations G11A, N28A and S139A which are all located on the chymotrypsin fold [21], [23], [24]. On the other hand, previously we also identified a mutant N214A, which owns a dramatically abolished activity but only slightly weakened dimerization [16]. Very unexpectedly, our determination of its crystal structure revealed that it still adopts a dimeric structure almost identical to that of the WT protease [25]. Nevertheless, the results with molecular dynamics (MD) simulations up to 30 ns unveiled that the N214A mutation triggers the dynamical instability of the catalytic machinery, with many key residues jumped to sample the conformations characteristic of the collapsed and inactivated state in R298A, thus establishing a dynamically-driven inactivation mechanism for the catalytic machinery of the SARS 3CLpro [25].

Here we studied another mutant we previously identified with three residues S284-T285-I286 mutated to Ala, designated as STI/A [16]. Interestingly, despite being far away from the active pocket, the mutations led to a 3.6-fold enhancement of the catalytic activity but only slightly enhanced dimerization. Here, our determination of its crystal structure reveals that STI/A still adopts the dimeric structure almost identical to that of the wild-type (WT), only with a slightly change of the extra-domain packing, which is similar to that observed in the more active form of the WT at high pH values (pH 7.6 and 8) [44]. As a consequence, to understand its underlying dynamical mechanism, we conducted 100-ns MD simulations for both WT and STI/A. Remarkably, the most dramatic changes in STI/A simulations are associated with the nano-channel. This change appears to slightly enhance the dynamic stability of the N-finger and helix A, which subsequently relay the dynamic effects to the contacted residues and finally to catalytic machinery. Ultimately, the key components composed of the catalytic machinery become more dynamically stable, thus rationalizing the enhancement of its catalytic activity. As coronavirus 3C-Like proteases share a similar enzymatic mechanism [1], [2], [26], our results would facilitate the development of strategies and agents by modulating protein dynamics to fight new coronaviruses whose outbreaks may occur in the future.

## Materials and Methods

### Accession Numbers

The structure coordinate of the STI/A mutant was deposited in Protein Data Bank, with PDB ID code of 3EA8.

### Generation of the recombinant STI/A mutant without any extra residue

Recently the extra N-terminal residues leftover from the cleavage of fusion proteins were demonstrated to significantly reduce the enzymatic activity [10], [18]. On the other hand, the SARS 3CLpro we previously studied had two extra residues Gly-Ser after the thrombin cleavage of the GST-3CLp fusion proteins [6], [16]. In order to remove the two extra residues, in the present study we transferred the gene encoding SARS 3CLp from the pGEX-4T-1 GST-fusion expression vector (Amersham Biosciences, GE Healthcare, Little Chalfont, UK) to the His-tagged pET28a vector. Subsequently, site-directed mutagenesis was utilized to shorten the thrombin cleavage sequence LVPR|GS (CTG GTT CCG CGT GGA TCC) engineered by the company to LVPR| (CTG GTT CCG CGT), which only constituted the thrombin cleavage site in conjunction with the first two N-terminal residues Ser-Gly of SARS 3CLp. Interestingly, thrombin cleaved this new site (LVPR|SG) very efficiently to release the authentic wild-type 3CLpro as well as N214A [25]. To produce STI/A mutant, site-directed mutagenesis was further used to mutate S284-T285-I286 to Ala as previously described [16], [25]. Benefited from this re-engineered cleavage site, we were able to produce both authentic WT 3CLpro [25] and its STI/A mutant without any extra residues from the fusion tag.

Experimentally, the His-tagged protease or mutants was expressed in *E. coli* strain BL21 (DE3) with induction by 0.4 mM isopropyl-1-thio-d-galactopyranoside (IPTG) under 20 degree overnight. The protease was obtained by in-gel cleavage with thrombin while the His-tag protein attached to the Nickel-NTA beads (QIAGEN), followed by a FPLC purification on a gel filtration column (HiLoad 16/60 Superdex 200). The pure protease was concentrated to 10 mg/ml and stored in the buffer (10 mM Tris-HCl, 10 mM DTT, pH 7.5). The molecular weight of the WT and N214A proteases were determined by a Voyager STR MALDI-TOF mass spectrometer (Applied Biosystems).

### Enzymatic activity assay and ITC characterization of dimerization

The enzymatic activities of the STI/A proteases were measured by a fluorescence resonance energy transfer (FRET)-based assay using a fluorogenic substrate peptide (Dabcyl-KTSAVLQSGFRKME-Edans) as previously described [15], [16], [25], [27]. Briefly, 1 ml reaction mixture contained 50 nM protease and fluorogenic substrate with concentrations ranging from 1 µM to 40 µM in a 5 mM Tris-HCl buffer with 5 mM DTT at pH 6.0, which is identical to the pH for crystallization. The enzyme activity was measured by monitoring the increase of the emission fluorescence at a wavelength of 538 nm with excitation at 355 nm using a Perkin-Elmer LS-50B luminescence spectrometer. The Km and kcat values were deduced from data analysis using Graphpad prism.

ITC (isothermal titration calorimetry) experiments were carried out to determine the monomer-dimer dissociation constants of the STI/A proteases as previously described [10], [25] using a Microcal VP ITC machine. Briefly, the protease samples and buffers were span at 13.3k rpm for one hour to remove the tiny particles and degas thoroughly. In titrations, the STI/A sample in 5 mM Tris-HCl buffer at pH 6.0 containing 5 mM DTT were loaded in the syringe, which was subsequently titrated into the same buffer in the cell. The obtained titration data with endothermic peaks were analyzed by the built-in Microcal ORIGIN software using a dimer-monomer dissociation model to generate the dissociation constants and the enthalpy changes.

### Crystallization, structure determination of the STI/A protease

The STI/A protease with a concentration of 10 mg/ml were crystallized in a 2 µl hanging drop using a condition identical to that previously reported except for a minor variation of the PEG6000 concentration [4], [18], [25]. After a three-day growth, a single crystal was picked up from the crystal clusters for diffraction with the cryoprotective buffer (20% glycerol with the mother liquid). The X-ray diffraction data were collected at Bruker X8 PROTEUM in-house X-ray machine.

The collected data sets were processed using the program HKL2000 in a resolution of 2.25 Å in the space group C2. Briefly, the phase determination for the mutant structure was done by the molecular replacement method using the WT SARS-CoV 3CLpro structure (PDB code: 2H2Z) [18] as the searching model by the program Phaser [28] in the program suite Phenix [29]. The Ala mutating residues (Ser284, Thr285, Ile286) were corrected in the program COOT [30]. The refinements and the addition of the solvent molecules of the models for the mutant were done in the program suite Phenix. The final model was analyzed by PROCHECK [31] and all figures were prepared using Pymol [32].

### Molecular dynamics (MD) simulations

The crystal structures of the WT (PDB code: 2H2Z) [18] and STI/A determined in the present study were selected as the initial models for molecular dynamics simulations as previously described [25]. The crystal structures PDB files were post-processed as previously described [33], [34].

The simulation cell is a periodic cubic box with a minimum distance of 10 Å between the protein and the box walls to ensure the protein would not directly interact with its own periodic images given the cutoff. The water molecules, described using the TIP3P model, were filled in the periodic cubic box for the all atom simulation, 6 Na^+^ ions were randomly placed to neutralize the charge in MD system for STI/A and WT dimers, Each system contained approximately 75,000 atoms.

Three independent 100-ns MD simulations for each protease were performed with the program GROMACS [35] with the AMBER-03 [36] all-atom force field. The long-range electrostatic interactions were treated using the fast particle-mesh Ewald summation method [35], with the real space cutoff of 9 Å and a cutoff of 14 Å was used for the calculation of van der Waals interactions. The temperature during the simulations was kept constant at 300 K by Berendsen's coupling. The pressure was held at 1 bar. The isothermal compressibility was 4.5*10^−5^ bar^−1^. The time step was set as 2 fs. All bond lengths including hydrogen atoms were constrained by the LINCS algorithm [37]. Prior to MD simulations, all the initial structures were relaxed by 500 steps of energy minimization using the steepest descent algorithm, followed by 100 ps equilibration with a harmonic restraint potential applied to all the heavy atoms of the protease.

### Calculation of enclosed volume

POVME program was used to calculate the enclosed volumes of the nano-channel and Thr25-Cys44 Leu-P2 substrate pocket [38]. Snapshots from each MD trajectory were taken at every 100-ps interval. A 3D-grid spacing was constructed around the Cβ of residues: Ser284, Thr285, Ile286 in the nano-channel, and Thr25 and Cys44 in the Leu-P2 substrate pocket. Grid points were included when they do not overlap with any atom of the studied residues or neighboring residues. The grid points were summed up to give the enclosed volume.

### Calculation of center of mass (COM), angle Θ

Heavy atoms of all atoms are used in the computation of center of mass: C, N, O and S. Hydrogen atoms are ignored. The COM is calculated at 1ps interval. For the angle Θ calculation, COM of the two chymotrypsin fold (residues 7–180) of both protomers and the extra-domain/DomainIII (residue 200–302) of each protomer. A vector is computed for each COM of Domain III to the COM of the 2 chymotrypsin-folds. The angle Θ is the angle between these 2 vectors.

### Correlation analysis

STI/A as well as N214A modulates the catalysis of the SASR 3CL protease without substantial conformational change, suggesting that the mutation effects are relayed to the catalytic machinery through dynamic allostery. So here we attempted to analyze the allosteric networks of WT, N214A and STI/A by a recently established approach called MutInf [39]. MutInf represents an entropy-based approach to analyze ensembles of protein conformers, such as those from molecular dynamics simulations by using internal coordinates and focusing on dihedral angles. In particular, this approach is even applicable in cases for which conformational changes are subtle, for example, because the coupling is mostly entropic in nature [39]. Briefly, this approach utilizes second-order terms from the configurational entropy expansion, called the mutual information, to identify pairs of residues with correlated conformations, or correlated motions, in an equilibrium ensemble [39]. In the present study, the normalized matrix values were used, and 0.3 was set up to be the threshold value to determine the pairs of highly correlated residues. The residue pairs with correlated values ≥0.3 make up the top 1% of the entire matrix.

## Results

### Activity and dimerization of the STI/A enzyme

By reengineering the thrombin cleavage site, we succeeded in obtaining the STI/A proteases without any extra residues leftover from the fusion tag. The enzyme was characterized by far-UV CD spectroscopy and its spectrum (spectra not shown) had no detectable difference from that with two extra residues Gly-Ser that we previously studied [16], thus indicating that the two extra residues have no detectable effect on the secondary structures.

**Figure 1:**
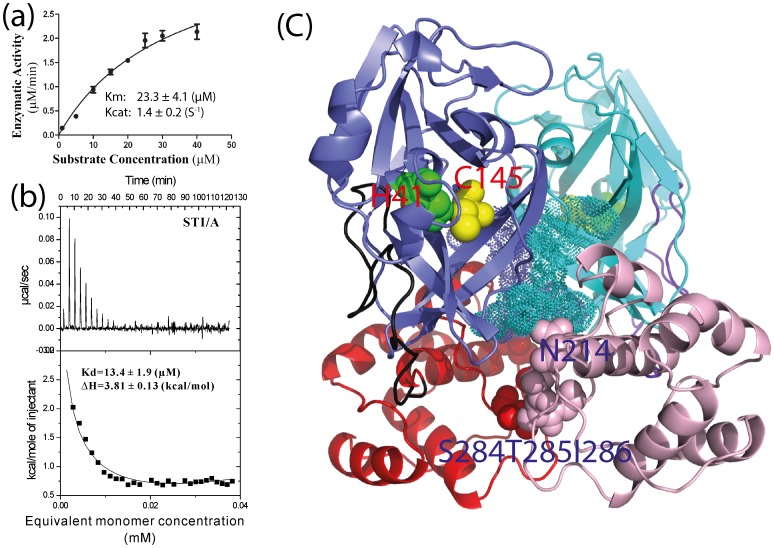
Enzymatic activity and dissociation constant of the dimer-monomer equilibrium. (a). Enzymatic activity of STI/A vs. substrate concentrations as measured by monitoring the increase of the emission fluorescence intensity at a wavelength of 538 nm. The Km and kcat values are presented. (b). The ITC dilution profiles for measuring the dissociation constant of the dimer-monomer equilibrium for STI/A. The Kd and ΔH values were obtained by fitting the ITC data with the built-in Microcal ORIGIN software. (c). Dimeric structure of the SARS 3CLpro with the catalytic folds colored in blue (protomer 1) and cyan (protomer 2); the extra domains in red (protomer 1) and lightpink (protomer 2), as well as the connecting loops in black (protomer 1) and purple (protomer 2). The catalytic dyad residue His41 is displayed as green sphere while Cys145 as yellow sphere. Mutation residues S284T285I286 and N214 are displayed as spheres and the N-finger residues Ser1-Fhe8 are displayed as dots.

By a fluorescence resonance energy transfer (FRET)-based assay, we have measured the enzymatic activities of the authentic STI/A proteases. As shown in Figure 1a, the STI/A protease has a Km value (23.3 µM) very similar to those previously reported on the authentic wild-type enzyme (24.2 µM) while Kcat of the STI/A protease has a 3.5-fold enhancements [25]. Here we further measured the Kd value of the STI/A dimerization by ITC to be 13.4 µM (Figure 1b), only slightly smaller than 21.4 µM for the authentic WT protease [25], indicating that the STI/A mutation only has a very small enhancement of the dimerization.

### Crystal structure of the STI/A enzyme

To gain insights into the structural consequence of the STI/A mutation, we determined its crystal structure at a resolution of 2.25 Å in C2 space group, with one molecule in the asymmetric unit. The R_work_ factor of the final models for STI/A mutant was 18.4%, while the R_free_ factor was 23.1%. Details of the data collection and refinement statistics are presented in Table S1.

**Figure 2:**
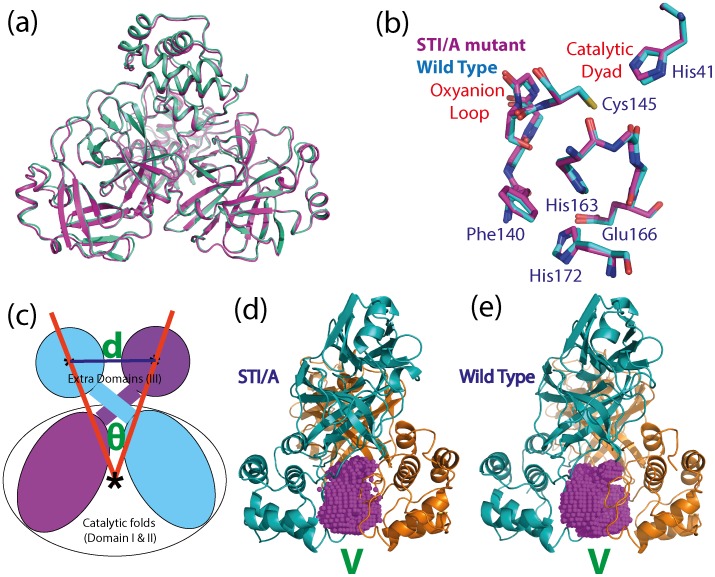
Crystal structure of the STI/A mutant. (a). Overall superimposition of the dimeric STI/A (violet) and WT (cyan; PDB code of 2H2Z (18) structures. (b) Superimpositions of the catalytically critical residues of STI/A (violet) and WT (cyan; PDB code of 2H2Z (18). (c). Diagram showing the distance (d) between the mass centers of two extra domains; and the angle (Θ) by the mass centers of two extra domains as well as the mass center of two chymotrypsin folds together (see Material and Methods for more details). The cavity volumes (V) of the nano-channels of STI/A (d) and WT (e) as represented by violet dots, which were calculated with the program: POVME (38).

In the electron density map of the STI/A mutant, all 306 residues are visible and it adopts the classic dimeric structure with the same packing of the two protomers as observed in all previously-determined dimeric structures of the coronavirus 3CLpro [1], [2], [4], [18], [19], [25], [40]–[45]. The dimeric STI/A structure is highly similar to those of the WT protease previously reported (Figure 2a). If compared to the WT crystal structure (2H2Z) [18] also determined at pH 6.0, with the authentic sequence in the same C2 space group [18], the dimeric RMS deviations are 0.86 and 0.77 Å respectively for the heavy (C, O, S and N) and backbone atoms (C, Cα, N (backbone-amide), carbonyl). Even if comparing at the individual protomer level, the RMS deviations reduce to 0.55 and 0.49 Å respectively for the heavy and backbone atoms, implying that the packing of two protomers slightly differs in the STI/A proteases. In the STI/A mutant, even for the key residues constituting the catalytic machinery including the catalytic dyad His41-Cys145, oxyanion-loop Phe140-Cys145, His163 and Glu166 critical for binding substrates; and Phe140, His172 in holding the substrate binding-pocket open, their backbones and side-chains are almost superimposable to those in the WT structure (Figure 2b). This observation implies that the enhancement of the STI/A activity cannot be readily rationalized only by the static structure. Furthermore, the mutation residues Ser284-Thr285-Ile286 are located on the extra domain and far away from the catalytic dyad His41-Cys145 (Figure 1c). We also examined the B-factors of STI/A and WT proteases but it appears extremely challenging to establish any precise correlation between the B-factors and catalytic activity. Therefore, the catalytic enhancement is most likely due to the change of the protein dynamics of the enzyme induced by the mutations as we previously demonstrated on the N214A mutant [25].

Intriguingly, the enzymatic activity of the wild-type 3CLpro has been previously found to be pH-dependent where the optimum activity is at pH 7.6 and the activity become much lower at pH 6.0 [4], [5], [18], [44]–[45]. Strikingly, upon pH changes, the extra domains in the dimeric enzyme appear to undergo a ‘rigid body rotation/movement’. Inspired by this, here we calculated the distances (d) between the centers of mass of two extra domains, as well as the angles (Θ) formed by two mass centers of each extra domains and the mass center of the two catalytic folds together (Figure 2c), which are 32.4 Å and 63.2 degree for the STI/A determined here at pH 6.0 (3EA7), 33.7 Å and 66.3 degree for the wild-type enzyme at pH 6.0 (2H2Z) [18], 32.6 Å and 63.3 degree for the wild-type enzyme at pH 7.6 (1UK3), and 32.7 Å and 63.7 degree for the wild-type enzyme at pH 8.0 (1UK2). This observation implies that the slightly tighter packing of the two extra domains is indeed associated with the higher catalytic activity.

Previously, based on a systematic mutagenesis study, we have proposed a nano-channel to be responsible for relaying the mutation effect from the extra domain to the catalytic machinery, which are mainly constituted by residues next to S284-T285-I286 on the extra domains as well as N-finger residues on the catalytic folds [16]. Because the mutation of STI to Ala with a small side chain results in a cavity, in STI/A the two extra domains come slightly closer as described above, and consequently the volume of the nano-channel (V) is 1293 Å^3^ in STI/A (Figure 2d), slightly smaller than that (1435 Å^3^) in WT (Figure 2e).

### Molecular dynamics (MD) simulations

Molecular dynamics simulation is a powerful tool to provide insights into the dynamic mechanism that underlies protein function [25], [46]–[50]. Therefore, we conducted 100-ns MD simulations for the wild-type (2H2Z) [18] and our present STI/A (3EA7) structures, both of which have been determined at pH 6.0 and in crystals of the space group C2.

**Figure 3:**
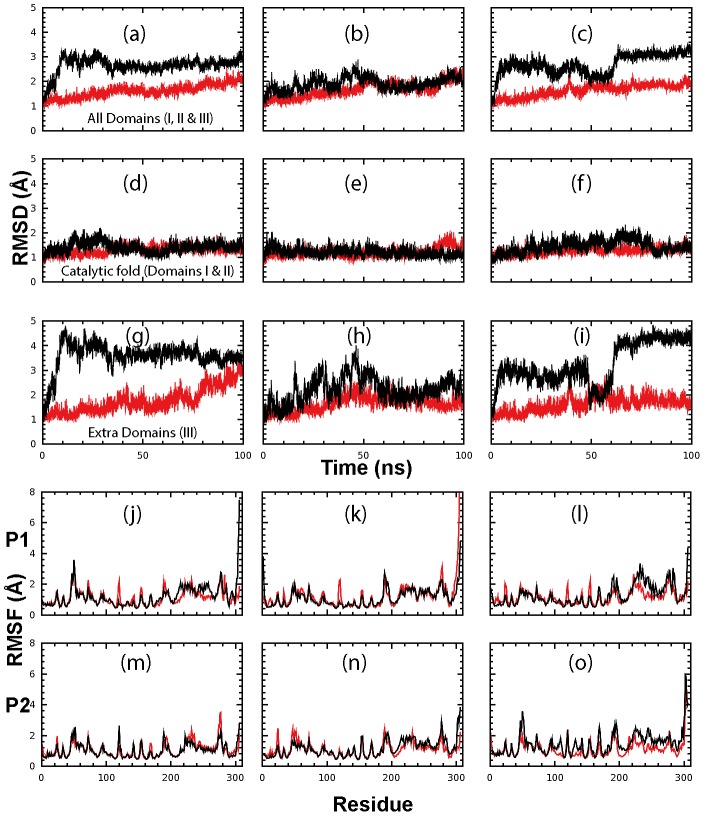
Overall dynamic behaviors in the MD simulations. Root-mean-square deviations (RMSD) of the Cα atoms (from their positions in the energy minimized structures) for three independent MD simulations of the dimeric STI/A (black) and WT (red) for the whole enzyme (a–c); catalytic fold (d–f); and extra domain (g–i). Root-mean-square fluctuations of the Ca atoms computed for three independent simulations for protomer A (j-l) and protomer B (m-o) of STI/A (black) and WT (red). Protomer A and B are denoted as P1 and P2 respectively.

Figure 3 presents the root-mean-square deviation (RMSD) of Cα atoms (from their positions in the energy minimized structures) for the dimeric WT and STI/A (a-c), their catalytic folds (domains I and II; d-f) and extra domains only (domain III, g-j). Interestingly, in the context of the dimeric forms, STI/A showed larger overall RMSD than WT, with average RMSD values over 100-ns simulations of 2.64±0.32, 1.90±0.26 and 2.68±0.42 Å for three separate simulations of STI/A; and 1.60±0.26, 1.66±0.31 and 1.63±0.25 Å for three simulations of WT. However, the averaged Cα RMSD values of the chymotrypsin folds (Domain I and II) of STI/A are only slightly larger than those of WT (Figures 3d-3f), with average RMSD values over 100-ns simulations of 1.40±0.21, 1.20±0.16 and 1.44±0.26 Å for three simulations of STI/A; and of 1.28±0.16, 1.17±0.17 and 1.25±0.15 Å for three simulations of WT. On the other hand, the extra domains of STI/A have dramatically higher fluctuations than WT in simulations as indicated by its higher RMSD values (Figures 3g-3i), with average RMSD values over 100-ns simulations of 3.57±0.54, 2.19±0.53 and 3.26±0.86 Å for three simulations of STI/A and of 1.75±0.51, 1.62±0.31 and 1.61±0.31 Å for three simulations of WT. These results clearly demonstrate that the larger RMSD values for the dimeric STI/A are mostly resulting from the motions of the extra domains. This premise is further evident from the root-mean-square fluctuation (RMSF) averaged over 100 ns for both STI/A and WT residues (Figures 3j-3o).

**Figure 4:**
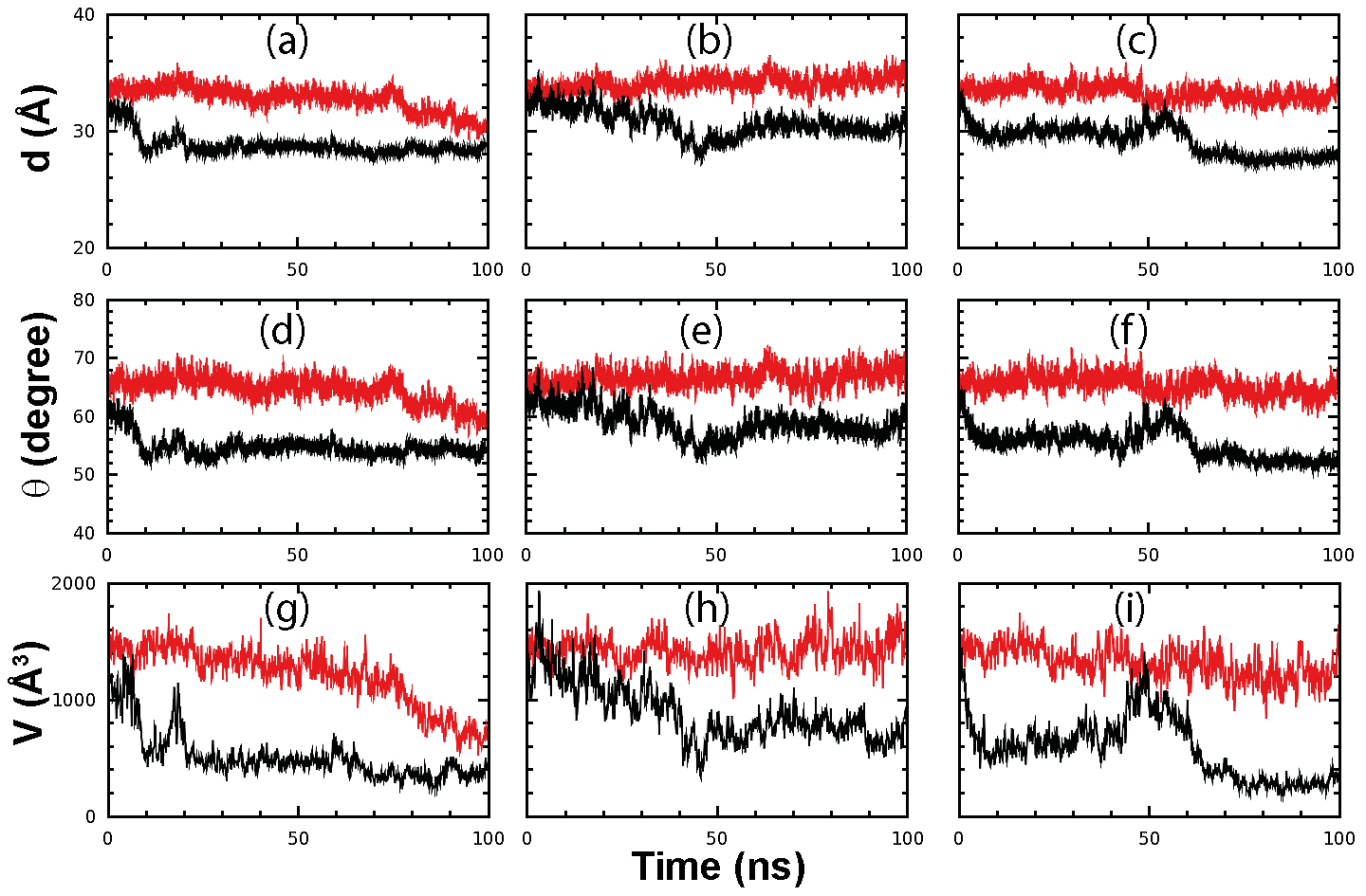
Changes of the packing interaction between two extra domains. Three separate time-trajectories of the distance d (a-c); angle Θ (see Figure 2d) (d-f) and volume of nano-channel (residue 284–286), V (g-i) of STI/A (black) and WT (red).

Indeed, for STI/A, the distance d (Figures 4a-c) and angle Θ (Figure 4d-f) as defined in Figure 2d have further reduced after ∼10 ns in simulations 1 and 3 and after ∼45 ns in simulation 2. For the average distance over 100-ns trajectories, STI/A has 28.77±0.90, 30.69±1.16 and 29.20±1.29 Å respectively for three simulations, while WT has 32.78±1.07, 34.12±0.62 and 33.33±0.656 Å respectively. For the average angle over 100-ns trajectories, STI/A has 54.78±1.781, 58.98±2.544 and 55.25±2.535 degree respectively for three simulations, while WT has 64.54±2.250, 66.65±1.461 and 65.43±1.614 degree respectively. These results indicate that in all three separate simulations, the two extra domains of STI/A become more tightly packed than those of WT. Consequently the volumes of the nano-channel significantly reduced for STI/A in all three simulations as compared to those of WT (Figures 4g-h), with average values of 512.62±212.52, 890.26±247.17 and 579.80±261.51 Å^3^ respectively for three simulations of STI/A, and of 1204.35±255.69, 1428.93±121.50 and 1319.94±143.21 Å^3^ respectively for WT.

We have also calculated the hydrogen bond occupancy of all simulations for both STI/A and WT, and Table 1 presents the hydrogen bond occupancies with the difference >5% between STI/A and WT. Interestingly, although the STI/A mutations are on the extra domain, only limited intra-domain hydrogen bonds show significant occupancy differences within the extra domain (Table 1). This suggests that similar to the tighter packing between two extra domains triggered by the high pH in WT [44], here the tighter packing induced by the STI/A mutations is also largely resulting from the ‘rigid body rotation/movement’ of the extra domains without significantly affecting the packing within the extra domains. Furthermore, the slight dynamic changes over the extra domains can also be transmitted to the catalytic fold, as exemplified by the significant changes of hydrogen bond occupancies between Asp295 and Thr111 (Table 1).

**Table 1:** Hydrogen Bond Occupancy for STI/A and WT with Significant Differences.

Donor				Acceptor				Average (%)		
Res No.	Name	Atom	Chain	Res No.	Name	Atom	Chain	STI/A	WT	(STI-WT)
**Extra Domain**										
276	MET	N	A	271	LEU	O	A	0.0	5.5	−5.5
276	MET	N	B	271	LEU	O	B	0.2	10.7	−10.5
278	GLY	N	B	285	THR	O	B	0.0	13.9	−13.9
230	PHE	N	A	226	THR	O	A	62.4	78.7	−16.3
230	PHE	N	B	226	THR	O	B	67.1	82.5	−15.4
257	THR	N	B	253	LEU	O	B	47.1	59.3	−12.2
299	GLN	N	A	296	VAL	O	A	13.1	36.1	−23
299	GLN	N	B	296	VAL	O	B	29.1	40.0	−10.9
										**−13.5±5.1**
**Extra Domain-Chymotrypsin Fold**										
111	THR	OG1	A	295	ASP	OD1	A	30.8	0.1	30.7
111	THR	OG1	B	295	ASP	OD1	B	34.3	15.7	18.6
									**24.7±8.6**	
**N-Fingers**										
4	ARG	NH1	A	290	GLU	OE2	B	29.3	44.6	−15.3
4	ARG	NH1	B	290	GLU	OE2	A	31.0	54.6	−23.6
4	ARG	NH2	A	137	LYS	O	B	14.1	24.4	−10.3
4	ARG	NH2	B	137	LYS	O	A	5.8	22.9	−17.1
5	LYS	NZ	A	288	GLU	OE2	A	31.6	20.6	11.9
5	LYS	NZ	B	288	GLU	OE2	B	21.3	14.0	7.3
7	ALA	N	A	125	VAL	O	B	83.6	67.1	16.5
									**−4.5±15.8**	
**Helix A at Domain I**										
10	SER	N	A	10	SER	OG	B	71.3	34.3	37.0
10	SER	N	B	10	SER	OG	A	61.5	33.8	27.7
11	GLY	N	B	14	GLU	OE1	A	93.3	32.8	60.5
11	GLY	N	A	14	GLU	OE1	B	62.4	72.8	−10.4
13	VAL	N	A	10	SER	O	A	87.6	48.1	39.5
15	GLY	N	B	11	GLY	O	B	53.2	9.1	44.1
95	ASN	ND2	A	15	GLY	O	A	72.7	67.0	5.7
95	ASN	ND2	B	15	GLY	O	B	75.6	63.9	11.7
									**27.0±23.2**	
**Asn28**										
28	ASN	ND2	A	117	CYS	O	A	27.5	10.4	17.1
28	ASN	ND2	B	117	CYS	O	B	32.4	18.0	14.4
28	ASN	ND2	A	145	CYS	O	A	87.6	30.5	57.1
28	ASN	ND2	B	145	CYS	O	B	78.0	56.0	22.0
										**27.7±19.9**
**Glu166**										
172	HIE	NE2	A	166	GLU	OE2	A	43.3	19.6	23.7
**Linker between Catalytic and Extra Domains**										
175	THR	OG1	A	176	ASP	O	A	16.9	26.5	−9.6
175	THR	OG1	B	176	ASP	O	B	27.2	39.2	−12.0
105	ARG	NH1	A	180	LYS	O	A	14.9	25.1	−10.2
105	ARG	NH1	B	180	LYS	O	B	12.1	34.3	−22.2
182	TYR	N	A	174	GLY	O	A	88.9	96.4	−7.5
182	TYR	N	B	174	GLY	O	B	89.4	95.6	−6.2
										**−11.3±5.7**
**Chymotrypsin Fold**										
19	GLN	NE2	A	26	THR	OG1	A	36.3	21.5	14.8
19	GLN	NE2	B	26	THR	OG1	B	59.5	0.0	59.5
19	GLN	NE2	A	119	ASN	O	A	5.5	20.3	−14.8
19	GLN	NE2	B	119	ASN	O	B	2.1	11.3	−9.2
21	THR	OG1	A	25	THR	O	A	0.0	8.8	−8.8
21	THR	OG1	B	25	THR	O	B	0.0	18.2	−18.2
22	CYS	N	A	25	THR	O	A	98.2	65.6	32.6
22	CYS	N	B	25	THR	O	B	98.5	63.1	35.4
25	THR	OG1	A	44	CYS	O	A	85.9	40.8	45.1
25	THR	OG1	B	44	CYS	O	B	75.9	52.8	23.1
25	THR	N	A	22	CYS	O	A	47.8	34.6	13.2
25	THR	N	B	22	CYS	O	B	43.4	24.4	19.0
26	THR	OG1	A	21	THR	OG1	A	74.4	62.3	12,1
26	THR	OG1	B	21	THR	OG1	B	85.0	25.8	59/2
113	SER	OG	A	127	GLN	OE1	A	95.5	84.4	11.1
113	SER	OG	B	127	GLN	OE1	B	92.7	46.1	46.6
116	ALA	N	A	124	GLY	O	A	92.7	75.6	17.1
117	CYS	N	B	147	SER	OG	B	80.2	65.8	14.4
121	SER	N	A	118	TYR	O	A	65.1	49.1	16.0
121	SER	N	B	118	TYR	O	B	61.6	56.0	5.6
135	THR	N	A	133	ASN	OD1	A	63.6	55.5	8.1
135	THR	N	B	133	ASN	OD1	B	61.6	54.7	6.9
148	VAL	N	A	115	LEU	O	A	63.0	20.8	42.2
									**−18.7±21.8**	

### Dynamic behavior of the catalytic dyad and oxyanion hole

**Figure 5:**
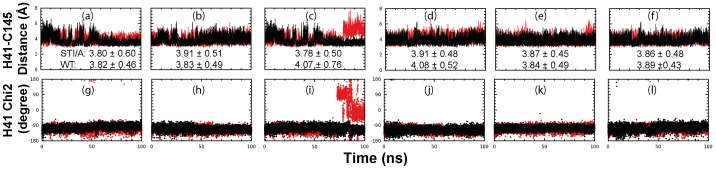
Dynamic behavior of the catalytic dyad. Three separate time-trajectories of the distance between NE2 of His41 and SG of Cys145 atoms of protomer A (a–c) and protomer B (d-f) for STI/A (black) and WT (red). Three separate time-trajectories of the Chi2 dihedral angle of His41 of protomer A (g-i) and protomer B (j-l) for STI/A (black) and WT (red). Protomer A and B are denoted as P1 and P2 respectively.

In the previously-determined crystal structures of SARS 3CLpro, the distance between NE2 of His41 and SG of Cys145 ranges from 3.6 to 3.9 Å. Furthermore, all previous MD simulations revealed that the dynamic stability of this distance is extremely critical for the stable formation of a hydrogen bond, which appears to be pivotal for maintaining the catalytic competency of the SARS 3CLpro [10], [23], [25], [51]–[53]. Also for the active WT enzyme, this distance has been previously demonstrated to be dynamically stable in MD simulations. Indeed, as in our previous simulations, the distances of both protomers of the WT were found to be dynamically stable for all 30 ns while those of the inactive N214A mutant became unstable and larger despite sharing a superimposable crystal structure to the WT [25]. In our current MD simulations of 100 ns, the distances between NE2 of His41 and SG of Cys145 of STI/A are: 3.80±0.60 Å, 3.91±0.51 and 3.78±0.50 Å respectively for three simulations of the protomer 1 (Figures 5a-5c), and 3.91±0.48, 3.87±0.45 and 3.86±0.48 Å respectively for the protomer 2 (Figures 5d-5f). In contrast, the distances in WT are: 3.82±0.46, 3.83±0.49 and 4.07±0.76 Å respectively for three simulations of the protomer 1, and 4.08±0.52, 3.84±0.49 and 3.89±0.43 Å respectively for the protomer 2. As a consequence, the distance of STI/A ranges from 3.78 to 3.91 Å while WT from 3.82 to 4.08 Å, suggesting that overall this distance is more dynamically stable in STI than that in WT. In particular, after 80 ns, in simulation 3 of the WT protomer 1, this distance becomes largely fluctuating (Figure 5c), while for STI/A, no such large fluctuation can be observed for all 100 ns. The Chi2 angles of His41 have similar dynamical behaviors (Figures 5g-5l) and the large fluctuation is also observed (Figure 5i).

One key component of the catalytic machinery of the SARS 3CLp is the substrate-binding pocket composed of six substrate sites, namely S1–S6, which correspond to the P1–P6 residues of the substrate [1], [2], [4], [23], [25]. The S1 substrate site is the most critical as it confers an absolute specificity for a Gln at the P1 position of the substrate. As such, maintaining the intact conformation of S1 substrate site is especially vital for catalysis. Briefly, the S1 substrate site can be divided into four parts: the oxyanion hole; His163; Glu166; Phe140 and its stabilizing elements [1], [2], [4], [23], [25]. The oxyanion hole refers to a structural element to donate two hydrogen bonds from main-chain amides of Gly-143 and Cys-145 to accommodate the main-chain oxygen of Gln-P1 as well as the tetrahedral intermediate during catalysis. Previously we demonstrated that in the inactive monomer R298A, the most distinguishable change is the collapse of the oxyanion hole as triggered by the chameleon formation of a short 3_10_-helix from a loop over residues Ser139-Phe140-Leu141 [22]. Remarkably, in our previous MD simulations, residues Ser139-Phe140-Leu141 of the WT maintained an extended active conformation while those of N214A frequently jumped to sample the 3_10_-helix conformation characteristic of the collapsed oxyanion hole [25].

In the present simulations of both STA and WT up to 100 ns, the three residues have similar dynamical behaviors in their backbone conformations (Figure S1), as well as the similar hydrophobic stacking interactions among Phe140, His163 and His172.

### Dynamic behavior of His163 and Glu166

The Glu-P1 substrate binding site includes essential substrate recognition residues: His163, Glu166 and His172. Extensive studies previously revealed that increased side-chain flexibility of His163 and Glu166 decreases the enzymatic activity [44], and mutation of Glu166 to alanine reduces the enzymatic activity by many folds [54]. Particularly these three residues were found to play a key role in the pH-dependent enhancement of the catalytic activity by both experiments and simulations [44]: the reduced contact between His163 and Glu166 allowed Glu166 to engage in hydrogen bonding with His172 and consequently adopted optimal conformations for substrate-binding at pH 7.6. In contrast, the increased contact between His163 and Glu166 decreased hydrogen bond between Glu166 and His172, and thus reduced the stability of the substrate pocket at pH 6 [44].

In our current 100-ns MD simulation, the backbone conformations of both His163 and Glu166 are indistinguishable between STI/A and WT. In STI/A, the distances of His163-Glu166 are: 7.97±0.32, 7.75±0.35 and 7.89±0.35 Å (with the average of 7.87 Å) for three simulations of protomer 1; 7.60±0.56, 8.31±0.31 and 8.18±0.50 Å (with the average of 8.03 Å) for protomer 2. In WT, the distances are: 8.02±0.42, 7.94±0.37 and 8.17±0.47 Å (with the average of 7.86 Å) for three simulations of protomer 1; 7.72±0.56, 8.05±0.34 and 7.81±0.44 Å (with the average of 8.04 Å) for protomer 2. Furthermore, the dynamic behavior of the His163-His172 distance is highly similar in both STI/A and WT (Figure S2a-S2f).

**Figure 6:**
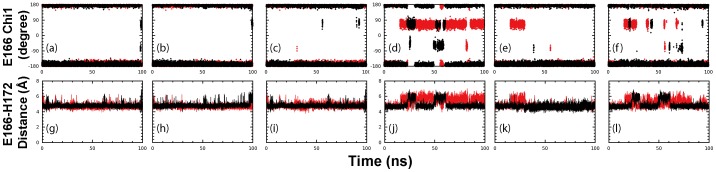
Dynamic behavior of the Glu166-His172 interaction. Three separate time-trajectories of the Chi1 dihedral angle of Glu166 of protomer A (a-c) and protomer B (d-f) for STI/A (black) and WT (red). Three separate time-trajectories of the distance between Glu166 and His172 of protomer A (g-i) and protomer B (j-l) for STI/A (black) and WT (red). Protomer A and B are denoted as P1 and P2 respectively.

Interestingly, STI/A showed enhanced dynamic stability in the Glu166 side-chain conformation (Figures 6a-6f) and smaller centroid distance between the side chains of Glu166 and His172 (Figures 6g-6l). The averaged centroid distances of Glu166 and His172 are: 4.80 Å and 4.81 Å respectively for two protomers of STI/A; and 4.73 and 5.13 Å respectively for two protomers of WT. The slight decrease in the distance between Glu166 and His172 in STI/A leads to an increased hydrogen bond occupancy between Glu166 and His172: 43.3% in STI/A versus 19.6% in WT (Table 1). Previously, the molecular mechanism for the pH-triggered activity enhancement has also been largely attributed to the increase in the Glu166 dynamic stability, which results from the different protonation states of His163 and His172 at higher pH [43]. This implies that the activity enhancement triggered by high pH and STI/A mutation may share overlapped allosteric pathways.

### Dynamic behavior of N-finger and α-helix A residues

In the dimeric structures, the N-finger residues Ser1-Ala7 of one protomer have extensive contacts with residues of another protomer which include those constituting the substrate-binding and the catalytic pocket as well as critical for the dimerization. On the other hand, the N-finger residues also make extensive contacts with the extra domains and are key component of the nano-channel [16]. As such, the dynamical changes of the extra domains may be relayed to other catalytic domain residues via the N-finger residues.

In the current MD simulations, the backbone conformations of the N-finger residues including Arg4 and Lys5 are very similar in both STI/A and WT. Nevertheless, the Arg4 side-chain conformations indicated by Chi1 is lopsided in STI/A: Arg4 sampled either of the two conformation clusters (30°<Chi1<90° and −30°<Chi1<−90°) in protomer 1 (Figures S3a-S3c) but sampled consistently only one conformation cluster (−30°<Chi1<−90°) in protomer 2 (Figures S3d-S3f). On the other hand, Arg4 in both protomers of WT sampled 3 conformation clusters. The side-chain conformation (Chi1) of Lys5 in STI/A prefers 2 conformation clusters (−180°<Chi1<−150° and 150°<Chi1<180°) (Figures S3m-S3r) while Lys5 in WT is restricted to one conformation cluster (−90°<Chi1<−30°). Interestingly, as shown in Table 1, in STI/A, Arg4 has lower hydrogen bond occupancies with Glu290 and Lys137, while Lys5 has higher hydrogen bond occupancies with Glu288.

The slight dynamical changes over the N-finger residues appear to affect the downstream helix A (Ser10-Gly15), which are mutually aligned at the dimer interface and engaged in inter-protomer hydrogen bonds and salt-bridges. Previously, the mutation of Gly11 to Ala has been shown to completely abolish the dimeric structure and lead to a collapsed catalytic machinery [23]. In the present simulations, although the backbone conformations of the helix A (Ser10-Gly15) are largely similar in the simulations of both STI/A and WT (Figure S4), the dynamic stability of the helix is largely enhanced, as evidenced by the significantly increased occupancies of the intra-residue Ser10 hydrogen bonds and those between Gly11 and Glu14, Ser10 and Val13, Gly11-Gly15, as well as Gly15-Asn95 (Table 1).

### Increased conformational stability of Asn28 and Thr25

**Figure 7:**
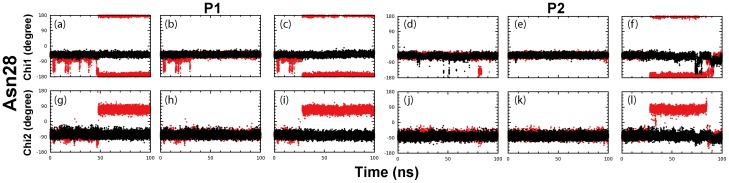
Dynamic behavior of Asn28. Three separate time-trajectories of the Chi1 dihedral angle of Asn28 of protomer A (a-c) and protomer B (d-f) for STI/A (black) and WT (red). Three separate time-trajectories of the Chi2 dihedral angle of Asn28 of protomer A (g-i) and protomer B (j-l) for STI/A (black) and WT (red). Protomer A and B are denoted as P1 and P2 respectively.

Previously, Asn28 has been identified to be a key component to mediate both catalysis and dimerization of the SARS 3CLpro via a long-range interaction network [7], [8], [21]. More specifically, it maintains the conformation integrity of and positioning of Cys145 (catalytic residue), Lys137-Phe140 (part of the oxyanion loop), Tyr126 and Cys117 through a hydrogen bond network composed of Asn28-Cys145, Asn28-Gly143, Asn28-Cys117 and Asn28-Gly120. Indeed, the mutation of Asn28 also led to a complete elimination of the dimeric structure and an inactivated and collapsed catalytic machinery [21]. In the present simulations, the backbone conformations of Asn28 are largely similar in STI/A and WT. However, the side-chain conformations of Asn28 as reflected by Chi1 (Figures 7a-7f) and Chi2 (Figures 7g-7l) exhibited an enhanced conformational stability in STI/A. On the other hand, in STI/A, Asn28 has significantly increased occupancies of the hydrogen bonds with Cys117 and Cys145 (Table 1). It appears that the enhanced stability of Asn28 contributes to the dynamical stability of the catalytic dyad (His41 and Cys145) (Figure 5).

**Figure 8:**
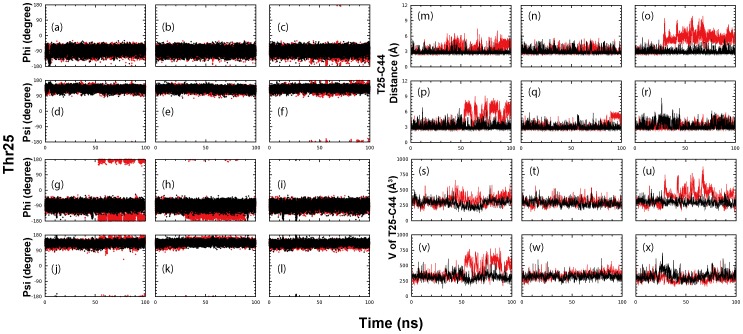
Dynamic behavior of Thr25 and Cys44. Three separate time-trajectories of Phi (a-c) and Psi (d-f) dihedral angles of Thr25 of protomer A; and Phi (g-i) and Psi (j-l) dihedral angles of Thr25 of protomer B for STI/A (black) and WT (red). Three separate time-trajectories of the distance between Thr25 and Cys44 of protomer A (m-o) and protomer B (p-r) for STI/A (black) and WT (red). Three separate time-trajectories of the volume enclosed by Thr25 and Cys44 of protomer A (s-u) and protomer B (v-x) for STI/A (black) and WT (red). Protomer A and B are denoted as P1 and P2 respectively.

Furthermore, we also observed an increased dynamical stability for residues of the Leu-P2 substrate pocket in the STI/A simulations. The Leu-P2 substrate binding site consists of Thr25, Leu27, Val42, Cys44, Thr47, Asp48, Met49, Tyr54, Leu164, and Met165 and these residues form a hydrophobic pocket that is receptive to a bulky side-chain such as Leu, Phe and Val [52]. Studies indicated that the physical dimensions or/and specific conformation of Leu-P2 substrate site determined both substrate binding specificity and consequentially the enzymatic catalytic rate. Thr25 and Cys44 lie at the edge of the Leu-P2 substrate binding site. The backbone and side-chain conformations of Cys44 are largely similar in both STI/A and WT simulations. Interestingly Thr25 shows a slightly enhanced dynamical stability of the backbone (Figures 8a-8l). Furthermore, the distance (Figures 8m-8r) and volume enclosed (Figures 8s-8x) between Thr25 and Cys44 have enhanced dynamical stability profiles in STI/A simulation: the averaged distance between Thr25/OG1 and Cys44/O is: 2.87±0.36 Å (protomer 1), 3.05±0.54 Å (protomer 2) for STI/A; and 3.63±0.92 Å (protomer 1), 3.40±1.00 Å (protomer 1) for WT. On the other hand, there is an approximate 2-fold increase in hydrogen bond occupancy between Thr25/OG1 and Cys44/O in STI/A simulations (Table 1). The significant fluctuations of the distance and volume between Thr25 and Cys44 in WT may result in an increased insertion of the methyl moiety of Thr25 side-chain into the cavity of Leu-P2 substrate site, thus impeding the docking of substrate into the substrate pocket or reduce the enzyme grip on the substrate during enzymatic catalysis.

### Other regions

We have also compared the dynamic behaviors of all other residues in STI/A and WT and found them to be very similar. Interestingly, although the STI/A mutations are on the extra domains, many residues in the chymotrypsin fold have the occupancies of their intra-domain hydrogen bond significantly affected (Table 1). First, the linker residues connecting the catalytic and extra domains have reduced hydrogen bond occupancies, which come from the rearrangement of the orientation between the catalytic and extra domains via ‘rigid body rotation/movement’ in SIT/A. Second, in STI/A simulations, except for the hydrogen bonds of Gln19-Asn119 and Thr21-Thr25 which have reduced occupancies, hydrogen bonds of other catalytic fold residues have significantly increased occupancies. These observations clearly indicate that the effects triggered by the STI/A mutations can be indeed transmitted to the catalytic fold and enhance the dynamic stability of the catalytic machinery, which ultimately leads to the increased catalytic activity of the STI/A mutant.

### Correlation analysis

**Figure 9:**
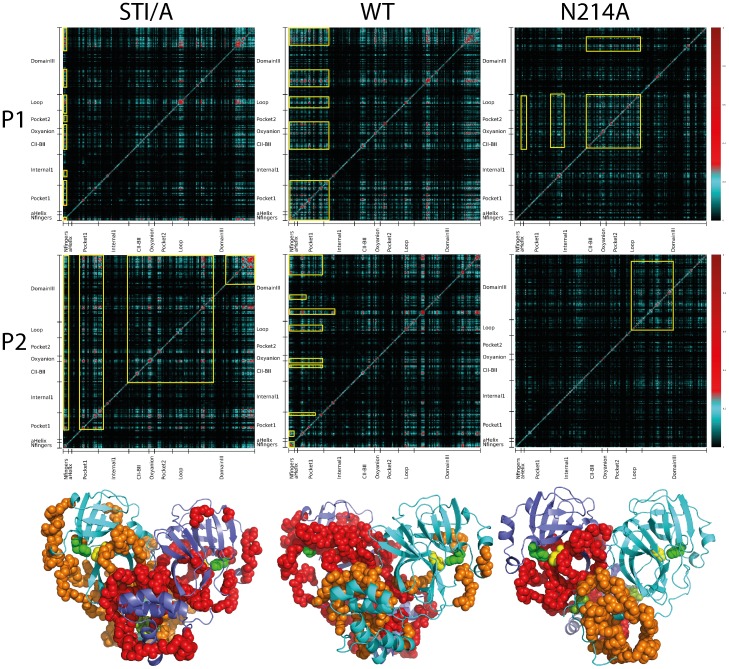
Networks of correlated motions in WT, STI/A and N214A. Mutual information matrixes calculated from the MD simulation data of WT, STI/A and N214A by MutInf (39); as well as the SARS 3CL protease structures with residues having significant correlation motion displayed in spheres which are colored in red if in the protomer 1; and brown in the protomer 2. The catalytic dyad residue His41 is displayed as green sphere while Cys145 as yellow sphere. The STI/A and N214A mutation residues are colored in splitpea. Yellow boxes in WT highlight the highly correlated motions between Phe3-Ser62 and: CII-BII (Leu115-Cys156), oxyanion loop (Ser139-Leu141), as well as extra domain (Asn214-Asn238, Ile281-Phe305). Yellow boxes in P1 of STI/A highlight the highly correlated motions of N-fingers with the other regions of the enzyme, similar to the WT while in P2 of STI/A highlight the expanded, highly correlated motions between the Domain III (Asn214-Asn238, Ile281-Phe305) and: substrate pocket (LeuP1), CII-BII (Leu115-Cys156), oxyanion loop (Ser139-Leu141), pocket 2 (Ser147-Thr175), loop region (Val186-Thr198). Yellow boxes in P1 of N214A highlight the correlated motions amongst residues in internal (Ile59-Arg105), CII-BII (Leu115-Cys156), oxyanion loop (Ser139-Leu141), catalytic dyad (Cys145) and the A Helix (Ser10-Glu14); while in P2 of N214A highlight the correlated motions within Domain III.

As seen in the mutual information profiles (Figure 9), in the protomer 1 of WT, fragments of both catalytic and extra domains have highly correlated motions, which include Phe3-Ser62 contaning the N-finger, helix A, Thr25, Asn28, Cys44 and catalytic dyad residue His41; Leu115-Cys156 containing CII-BII residues, oxyanion loop Ser139-Leu141, and catalytic dyad residue Cys145; Val186-Thr198 within the loop connecting the catalytic and extra domain; Asn214-Asn238 containing Asn214; and Ile281-Phe305 containing S284-T285-I286. These fragments cover all residues which have been identified to be critical for dimerization and catalysis previously as well as in the present study, which constitute a correlated network over the whole protease (Figure 9). Interestingly, here the loop residues Val186-Thr198 are revealed to be a key component of this network. Previously their role in dimerization and catalysis has been mostly unknown and thus it is worthwhile to experimentally characterize in the further. Furthermore, although the correlation pattern in the protomer 2 of WT remains largely similar, the significantly correlated pairs of residues slightly changed, thus leading to the less correlation of some catalytic domain residues (Figure 9).

Strikingly, although the N214A mutation significantly provoked the dynamics of the whole protease [25], the mutual information profiles reveal that it globally decouples the correlation of the paired residue motions (Figure 9). As a consequence, the first half of the catalytic fold which hosts His41, one of the catalytic dyad residues, loses the significant correlation to the rest of the protease in both protomers. The results on the correlation analysis of both WT and N214A strongly suggest that the networks to correlate the motions over the whole protease are essential for implementing its catalytic action, and the dynamic perturbation onto a key component even without detectable conformational change is sufficient to dramatically inactivate the catalytic machinery by disrupting the correlation network. On the other hand, for STI/A, the difference of the mutual information profiles between STI/A and WT is relatively small. In the protomer 1 of STI/A, although the correlated motions of some pairs of residues become less significant as compare to those in WT, the correlated motions become significantly increased for residues Gly2-Arg4 with many other residues distributed over the whole protease. In the second protomer of STI/A, the correlated motions over the majority of the residue pairs become enhanced as compared to those of WT. As such, it is not straightforward to attribute these changes to the activity enhancement in STI/A. It appears that unlike the N214A mutation which decouples the global correlated motions, thus inactivating the catalytic machinery, the STI/A mutation enhances the catalytic activity by the specific allosteric pathways which manifest as the altered correlation patterns.

## Discussion

Due to the severity of the worldwide SARS epidemic, the SARS-CoV received extremely-intense research efforts worldwide immediately after its outbreak. The SARS 3CLpro not only represents a promising target for developing therapeutic agents, but also serves as an excellent model for understanding how the evolutionarily-acquired non-catalytic domains mediate the enzymatic mechanisms. Previously, it has been found that by acquiring additional non-catalytic domains during evolution, many enzymes gain altered catalytic mechanisms or/and be connected to cellular signaling networks [55]–[56]. Indeed, upon acquiring the C-terminal extra domain, SARS 3CLpro suddenly needs the dimerization to activate its catalytic machinery. Previous studies have uncovered that the monomeric structures of the SARS 3CLpro have the same collapsed catalytic machinery, regardless of being triggered by G11A, N28A or S139A mutations on the catalytic domains [21], [23], [24], or by R298A on the extra domain [22]. This suggests that the dimerization is commonly controlled by a structurally-allosteric network composed of residues of both catalytic and extra domains. With MD simulations, we have shown that the collapsed catalytic machinery was not only structurally-distinguishable from, but also dynamically well-separated from the activated state of SARS 3CLpro [25]. Remarkably, despite having almost the same three-dimensional structure as WT, in MD simulations, the dimeric but inactive N214A mutant frequently jumped to sample the conformations of the collapsed state, thus leading to the proposal of the “dynamically-driven inactivation” for the catalytic machinery of SARS 3CLpro [25]. This observation implies that the mutation effect of N214A has been relayed to inactivating the catalytic machinery through the dynamic allostery.

**Figure 10:**
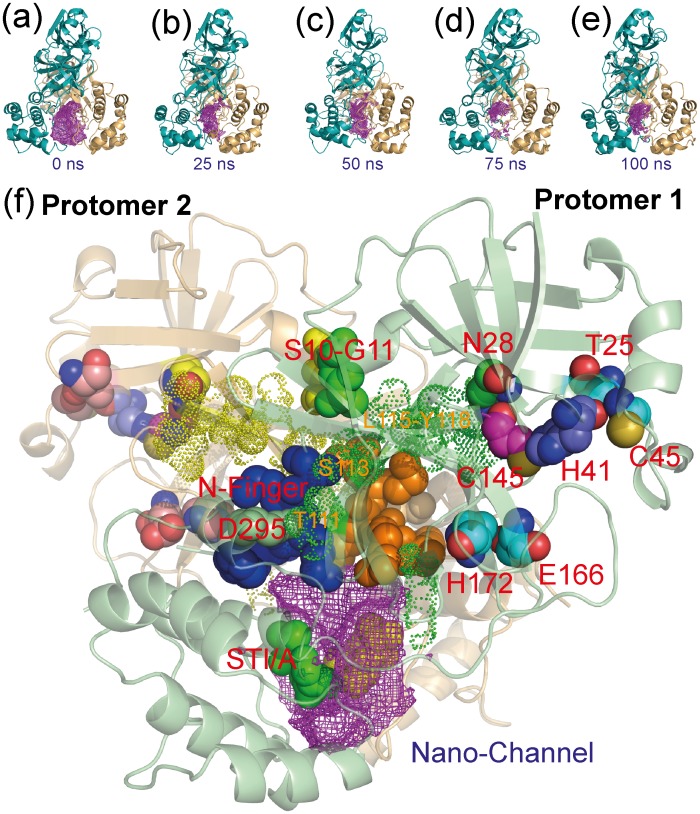
Transmission of the STI/A mutation effects on the extra domains to the catalytic machinery by the dynamically-driven allostery. (a)-(e). The cavity volumes of the nano-channel of STI/A in the first simulation at 0, 25, 50, 75 and 100 ns. (f). The crystal structure of STI/A with key residues having relevant dynamical changes displayed and labeled. The cavity is represented by the violet mesh.

Here we studied another mutant STI/A with mutations on the extra domain, which has a significantly-increased activity but only slightly-enhanced dimerization. The successful determination of its crystal structure indicates that STI/A still adopts the dimeric structure almost identical to that of WT, only with slightly tighter packing between two extra-domains due to the cavity created from replacing Ser284-Thr285-Ile286 with Ala (Figure 2a). In particular, the key residues constituting the catalytic machinery are almost superimposable to those of WT (Figure 2b). We thus hypothesized that the activity enhancement may be mostly resulting from the dynamic allostery, as we previously observed in the N214A mutant [25]. Consequently we conducted 100-ns MD simulations for both STI/A and WT, which represent the longest simulations reported for 3CLpro so far. An exhaustive analysis of MD results revealed that STI/A and WT have very similar dynamical behaviors for most residues. Nevertheless, in the MD simulations, STI/A did show some dynamical behaviors different from those in WT. The most significant change in STI/A is that in the simulations the two extra domains became further tightly packed in STI/A, mostly driven by the hydrophobic interactions by Ala284-Ala285-Ala286-Leu287. This led to a dramatic reduction of the volume of a nano-channel constituted by residues of both catalytic and extra domains (Figures 10a-10e), which we previously proposed to relay the perturbation on the extra domains to the catalytic machinery [16]. The volume reduction in the nano-channel triggered slight changes of dynamics of some nano-channel residues (Figure 10f), in particular the N-finger residues (Figure S3), which are also reflected by the redistribution of the hydrogen bond occupancy (Table 1). Interestingly the Helix A residues also become slightly more stable as evident from their backbone conformations (Figure S4) and increased hydrogen-bond occupancy for the residues Ser10 and Gly11 (Table 1). The dynamic changes over N-finger and helix A appears to be further transmitted to residues over the BII and CII beta-strands including Thr111, Ser113, Leu115-Tyr118 and Ser123 (Figure 10f), which ultimately lead to the enhanced dynamic stability of residues constituting the catalytic machinery, such as Asn28, Thr25 and Cys45; Glu166 and His172; as well as the catalytic dyad His41-145. The enhanced dynamical stability of these residues appears to be responsible for the increased activity of STI/A. Indeed, previously high catalytic activity of some enzymes has been correlated with their high stability [57]–[59] and in particular, a lipase mutant with higher catalytic activity has been characterized to have active sites of higher dynamical rigidity by both MD simulation and experimental studies [59].

Amazingly, the residues involved in the dynamic transmission in STI/A have been previously characterized to be also critical for maintaining dimerization. For example, the N-finger [20], Gly11 [23] and Asn28 [21] residues are particularly important and mutation/deletion of them has been shown to abolish the dimeric structures. This implies the existence of a correlated interaction network, which is constituted by interactions of residues of both catalytic and extra domains [21], [22]. Indeed, our correlation analysis by MutInf [39] reveals that such a global network of the correlated motions does exist in the WT protease, whose components include all residues identified to be critical for its dimerization and catalysis previously as well as in the current study (Figure 10). Most remarkably, the N214A mutation appears to inactivate the catalytic machinery at least partly by decoupling the correlation of the network components while the STI/A mutation enhance the activity by altering the correlation pattern. Indeed, the inactive N214A mutant does have a slightly weakened dimerization [25] while the more active STI/A mutant has a slightly enhanced dimerization, which strongly supports the proposal that the specific structured crowding can have significant effects on enzymatic catalysis through mediating protein dynamics and their correlations [49].

The results thus decipher a global correlation network in the SARS 3CL protease which not only couples the dimerization and catalysis by the structural allostery as previously demonstrated [21]–[24], but also by the dynamic allostery. Previously, the dynamic changes triggered by mutations have been extensively demonstrated to mediate enzymatic catalysis by both experiments and MD simulations [47]–[49], [60]–[64]. However, it still remains rare to find that the catalytic machinery can be dynamically modulated by the mutations on the evolutionarily-gained non-catalytic domain, which are also far away from the active center. To the best of our knowledge, the SARS 3CLpro appears to be the first example that without having significant structural change over the active pocket, the mutation perturbations on the evolutionarily-acquired non-catalytic domain can be relayed by the dynamic allostery into manifesting opposite catalytic effects: inactivation of catalysis in N214A and enhancement in STI/A. This proposition implies that in addition to the structural allostery, the dynamic allostery also plays key roles in controlling catalysis, which may extensively exists in other enzymes.

New coronaviruses including human beta-coronavirus 2c EMC/2012 (HCoV-EMC) may cause great threats to human health in the near future [1], [2], [26]. However, one unsolved challenge to fight against them is to design inhibitors for the 3CL proteases with high specificity. Based on the present results, a promising avenue may be opened up to design very specific inhibitors to disrupt the global networks of the correlated motions through targeting the network components unique for each 3CL protease.

## Supporting Information

Figure S1 **Dynamic behavior of the oxyanion loop residues.** Ramachandran plots of the residues Ser139-Phe140-Leu141 for STI/A (black) and WT (red). Protomer A and B are denoted as P1 and P2 respectively.
(TIF)

Figure S2 **Dynamic behavior of the His163-His172 interaction.** Three separate time-trajectories of the centroid distances between the aromatic rings of His163 and His172 of protomer A (a-c) and protomer B (d-f) for STI/A (black) and WT (red). Protomer A and B are denoted as P1 and P2 respectively.
(TIF)

Figure S3 **Dynamic behavior of the N-finger residues Arg4 and Lys5.** Three separate time-trajectories of the Chi1 and Chi2 dihedral angles of Arg4 and Lys5 for STI/A (black) and WT (red). Protomer A and B are denoted as P1 and P2 respectively.
(TIF)

Figure S4 **Dynamic behavior of the Helix A residues.** Ramachandran plots of the residues Ser10-Gly11-Lys12-Val13-Glu14-Gly15 for STI/A (black) and WT (red). Protomer A and B are denoted as P1 and P2 respectively.
(TIF)

Table S1 **Data collection and refinement statistics for the STI/A mutant.**
(DOCX)
