# Supplementary figures and images for: Dynamically-Driven Enhancement of the Catalytic Machinery of the SARS 3C-Like Protease by the S284-T285-I286/A Mutations on the Extra Domain
Source: PLoS One. 2014 Jul 18;9(7):e101941. doi: 10.1371/journal.pone.0101941 (PMC4103764; doi:10.1371/journal.pone.0101941)

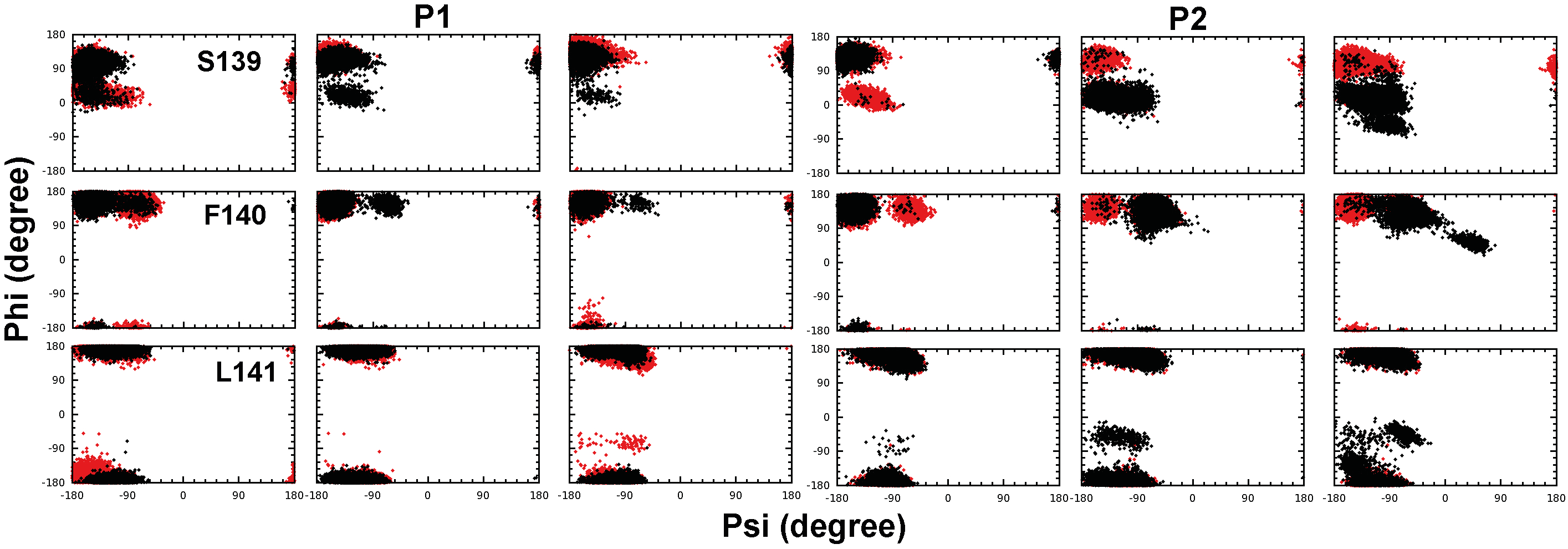

Supplement: Figure S1 — Dynamic behavior of the oxyanion loop residues. Ramachandran plots of the residues Ser139-Phe140-Leu141 for STI/A (black) and WT (red). Protomer A and B are denoted as P1 and P2 respectively. (TIF) [file pone.0101941.s001.tif]

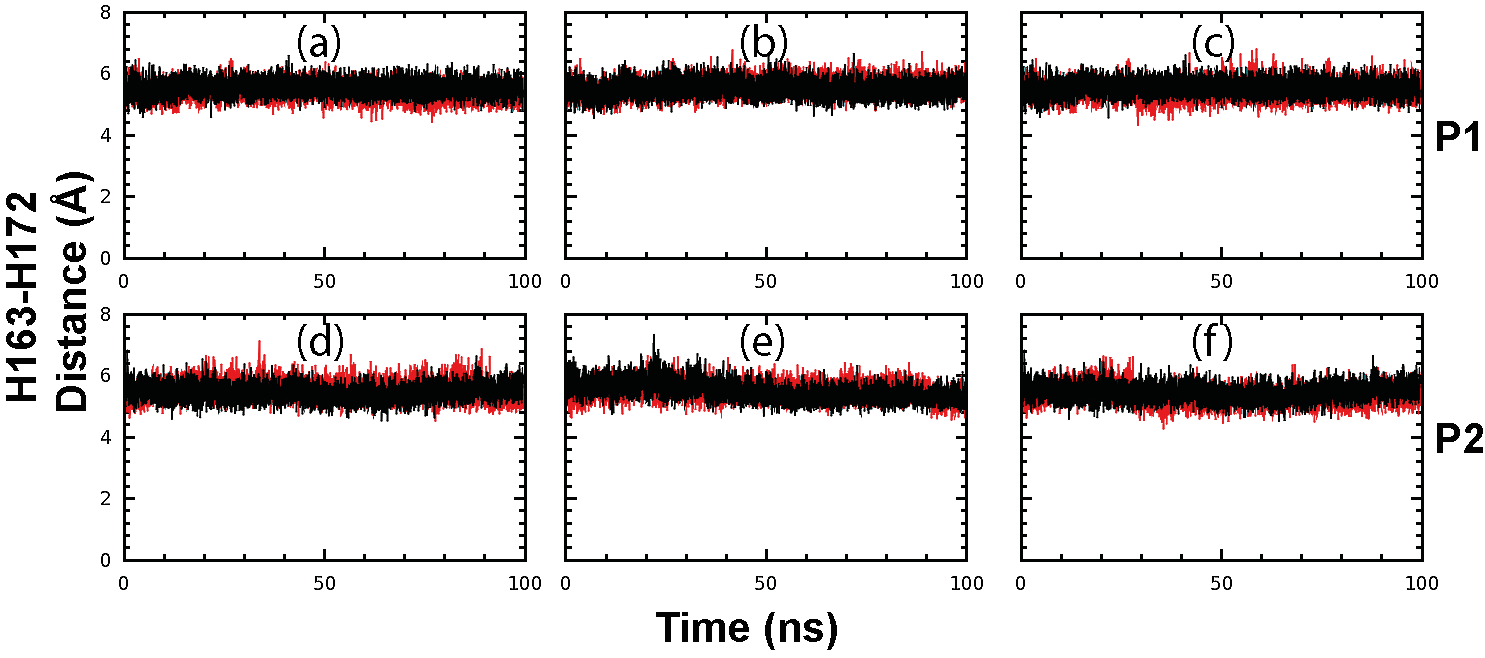

Supplement: Figure S2 — Dynamic behavior of the His163-His172 interaction. Three separate time-trajectories of the centroid distances between the aromatic rings of His163 and His172 of protomer A (a-c) and protomer B (d-f) for STI/A (black) and WT (red). Protomer A and B are denoted as P1 and P2 respectively. (TIF) [file pone.0101941.s002.tif]

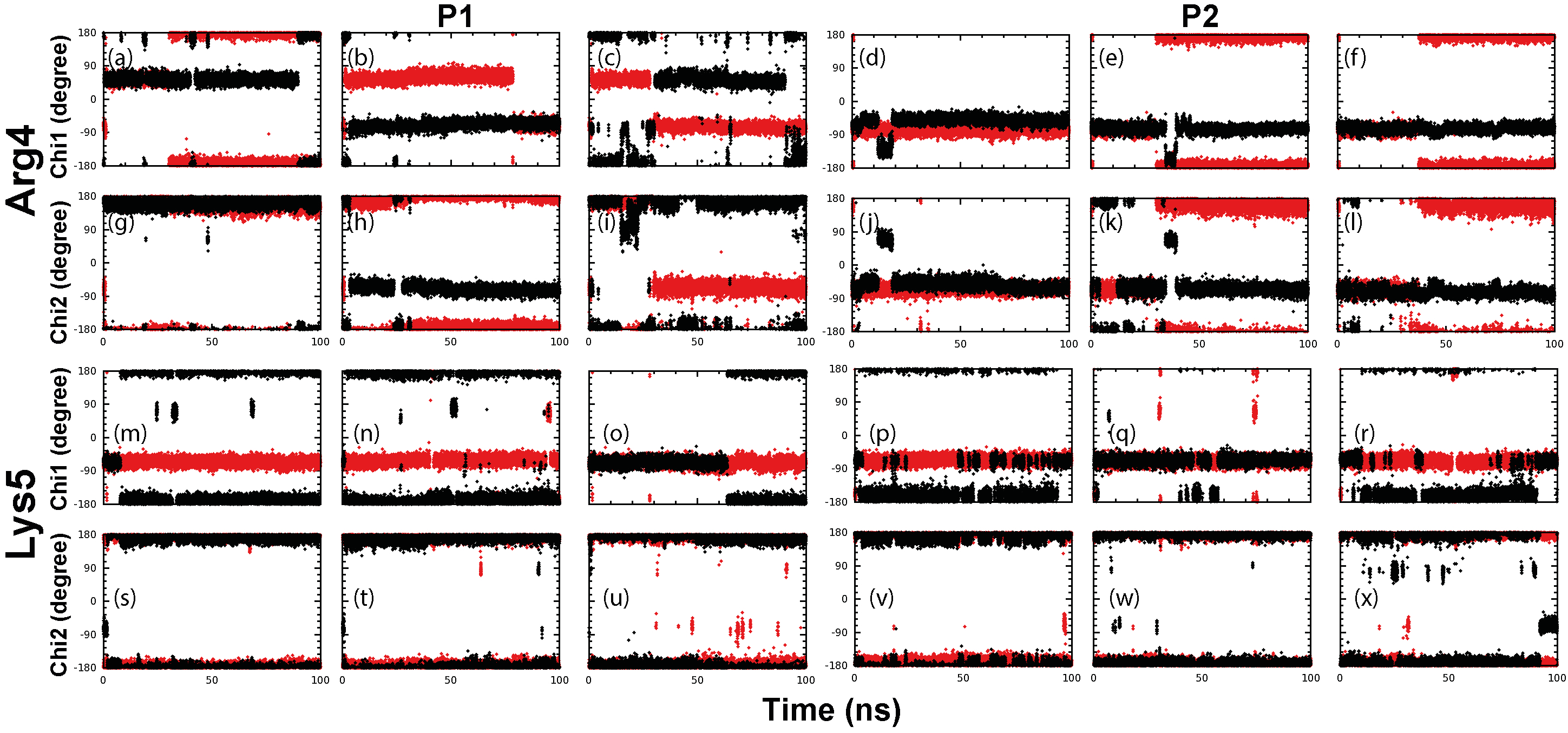

Supplement: Figure S3 — Dynamic behavior of the N-finger residues Arg4 and Lys5. Three separate time-trajectories of the Chi1 and Chi2 dihedral angles of Arg4 and Lys5 for STI/A (black) and WT (red). Protomer A and B are denoted as P1 and P2 respectively. (TIF) [file pone.0101941.s003.tif]

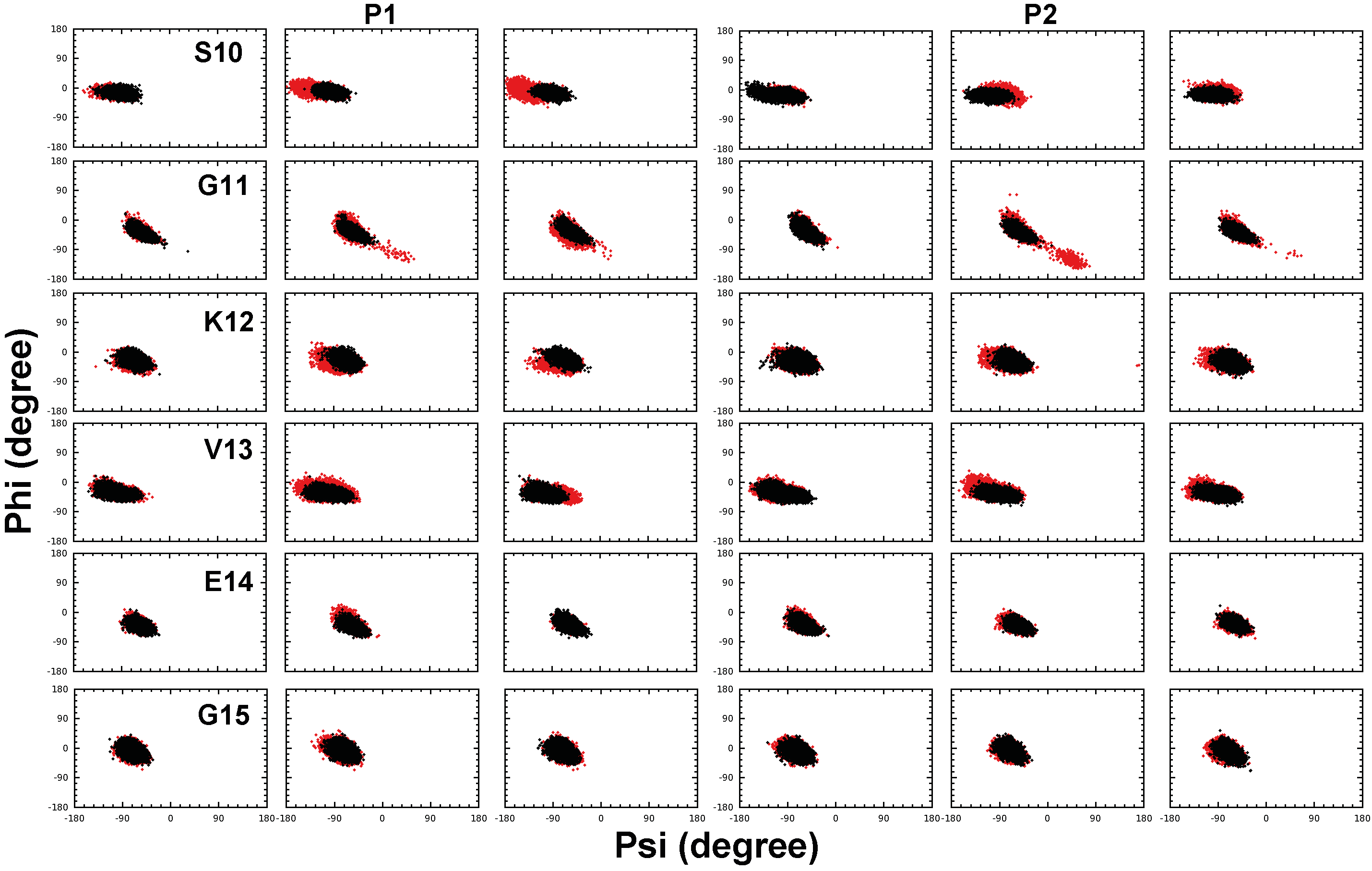

Supplement: Figure S4 — Dynamic behavior of the Helix A residues. Ramachandran plots of the residues Ser10-Gly11-Lys12-Val13-Glu14-Gly15 for STI/A (black) and WT (red). Protomer A and B are denoted as P1 and P2 respectively. (TIF) [file pone.0101941.s004.tif]
