# Supplementary material for: Dynamically-Driven Enhancement of the Catalytic Machinery of the SARS 3C-Like Protease by the S284-T285-I286/A Mutations on the Extra Domain
Source: PLoS One. 2014 Jul 18;9(7):e101941. doi: 10.1371/journal.pone.0101941 (PMC4103764; doi:10.1371/journal.pone.0101941)
Supplement: Table S1 — Data collection and refinement statistics for the STI/A mutant. (DOCX) [file pone.0101941.s005.docx]

**Table 1**: **Data collection and refinement statistics for the STI/A mutant**

|  | SARS 3CLpro triple mutant (STI/A) |
| --- | --- |
| **PDB ID** | 3EA8 |
| **Data collection** |  |
| Space group | C2 |
| No. of molecule in ASU | 1 |
| Wavelength (Å) | 1.5418 |
| Resolution (Å) | 42.59- 2.25 (2.36-2.25) |
| Cell dimensions |  |
| *a* (Å) | 107.15 |
| *b* (Å) | 82.56 |
| *c* (Å) | 52.75 |
| σ (°) | 90 |
| β (°) | 106.49 |
| γ (°) | 90 |
| Observed reflections | 116159 |
| Unique reflections | 20930 (2307) |
| *R*_sym_ or *R*_merge_ | 0.067 (0.301) |
| *I* / σ*I* | 11.38 (3.28) |
| Completeness (%) | 100 (99.7) |
| Redundancy | 5.53 (4.77) |
|  |  |
| **Refinement** |  |
| Resolution (Å) | 49.59-2.25 (2.36-2.25) |
| No. reflections | 19120 (2265) |
| *R*_work_ | 0.1839 (0.1988) |
| No. reflections | 18157 (2150) |
| *R*_free_ | 0.2314 (0.2624) |
| R.m.s. deviations |  |
| Bond lengths (Å) | 0.007 |
| Bond angles (°) | 1.063 |
|  |  |
| **Ramachandran plot** |  |
| Favored, % | 90.9 |
| Allowed, % | 7.9 |
| Generously allowed, % | 0.8 |
| Disallowed, % | 0.4 |

One crystal was used for data collection. Values in parentheses are for highest-resolution shell.
